# Clinical Presentation and Laboratory Diagnostic Work-Up of a Horse with Tick-Borne Encephalitis in Switzerland

**DOI:** 10.3390/v13081474

**Published:** 2021-07-28

**Authors:** Nathalie Fouché, Solange Oesch, Ute Ziegler, Vinzenz Gerber

**Affiliations:** 1 Swiss Institute of Equine Medicine, University of Bern, and Agroscope, 3012 Bern, Switzerland; solange.oesch@vetsuisse.unibe.ch (S.O.); vinzenz.gerber@vetsuisse.unibe.ch (V.G.); 2 Institute of Novel and Emerging Infectious Diseases, Friedrich-Loeffler-Institut, 17493 Greifswald, Insel Riems, Germany; ute.ziegler@fli.de

**Keywords:** ataxia, equine, neurologic disease, viral infection

## Abstract

Tick-borne encephalitis is an important viral tick-borne zoonosis in Europe and Asia. The disease is induced by tick-borne encephalitis virus (TBEV). This report describes a 16-year-old Warmblood gelding presenting with sudden onset of lethargy, ataxia, and muscle fasciculations on the nostrils, the lips, and the eye lids as the most important clinical findings. The horse further had a mild facial nerve paralysis with drooping of the right upper and lower lip. Diagnosis was based on paired serum samples using TBEV-ELISAs revealing high serum IgM in the first sample with normal IgM in the second sample and an increase in serum IgG and neutralizing antibodies, indicating acute and recent infection. TBEV was confirmed by a virus-neutralization test, revealing a fivefold increase in antibodies 32 days after of the onset of clinical signs. Although the specific PCR on cerebrospinal fluid (CSF) was negative, TBEV-specific IgG and IgM were identified in the CSF of the horse. Treatment consisted of anti-inflammatory and anti-oxidative treatment and the horse recovered with a mild drooping of the right nostril as the only remaining clinical sign. TBEV infection is a potential differential diagnosis of neurological disease in horses living in endemic areas and this is the first report to describe the diagnostic criteria in a horse as recommended in humans with suspected TBEV infection.

## 1. Introduction

Tick-borne encephalitis (TBE) is an arboviral infection with tick-borne encephalitis virus (TBEV) causing neurological signs in humans and animals. Transmission of TBEV mostly occurs via infected ticks [1], although rare transmission by unpasteurized dairy products has been reported [2]. The incidence of the disease in humans reportedly increases in Europe owing to an expansion of endemic areas and prolongation of the tick activity season [3,4], which may increase the risk for clinical disease in horses.

The literature on the occurrence of TBE in horses includes epidemiological studies and reports of non-specific and neurological signs associated with suspected TBEV infections [5,6,7,8,9,10,11,12,13]. Most studies report seroprevalence data of TBEV in different areas of Europe and discuss the potential importance of the horse as a sentinel for the disease [7,10,14]. Although the occurrence of TBEV in different parts of Switzerland is well documented [15,16,17,18,19,20,21], the seroprevalence of TBEV in the equine population in this country has not been investigated to date. In other European countries, seroprevalence in horses ranged from 2.9 to 37.5%, depending on the geographical location, age structure, and management practices of the study population [5,7,9,10,12,13,22]. Although neurological disease in combination with seropositivity to TBEV would be expected in a horse suffering from TBE, an increase in IgG levels alone is a reflection of virus exposure and not a confirmation of acute infection.

Clinical signs associated with suspected infections in horses are described in several reports [6,11,12,13], but postmortem confirmation of infection in a horse with overt neurological signs was only performed in one early case study [11]. This is in contrast to other species, specifically dogs, where postmortem confirmation of TBE has been described repeatedly [23,24,25,26].

Laboratory findings of equine cases with suspected TBE have previously been reported [6,12,13]. Nevertheless, to the best of the author’s knowledge, cerebrospinal fluid (CSF) analysis, serum and CSF IgM and IgG levels, and IgG seroconversion, as recommended for the in vivo confirmation of the diagnosis of TBE in humans [2], have not been reported in horses to date.

In this report, we describe the diagnostic tests to confirm the ante-mortem diagnosis in a horse with clinical signs compatible with TBE. Laboratory testing included the confirmation of acute disease using ELISA tests for IgG and IgM in serum and CSF and excluded potential cross-reaction with other flaviviruses using a serum neutralization test as previously recommended [2,27,28].

## 2. Case History and Clinical Findings

A 16-year-old Holsteiner gelding was referred for evaluation of acute onset ataxia and lethargy. Furthermore, the horse showed muscle tremors and was mildly sweating. The gelding was treated with metamizol and dexamethasone prior to referral to the equine hospital on the same day.

At home, the horse was kept in a stable with daily access to pasture with other horses in a rural area. The gelding never travelled abroad nor within Switzerland. According to the owner, except for the winter season, the horse was regularly affected by tick bites.

Upon presentation, the horse was in good general condition. Vital parameters were within normal limits apart from an increased heart rate (48 beats per minute) after transportation. During the first clinical examination, the animal was bright and responsive with normal mentation. There were no cranial nerve deficits apart from a questionable mild facial nerve paralysis with drooping of the right upper and lower lip. An asymmetry of the temporal muscle with a slightly decreased tone on the right side was also noted. The horse showed fine muscle fasciculations and tremors on the nostrils, lips, and eye lids. The cervico-fascial reflex was absent bilaterally. The gelding was eating, drinking, defecating, and urinating normally. Passive and active ventral, dorsal and lateral neck movements, tail and anal tone, cutaneous trunci (panniculus), and perineal reflexes were normal, and no proprioceptive deficits were observed. In the first 24 h of hospitalisation, the horse displayed periods of severe lethargy, during which incoordination with a drift towards the right side was observed when the horse walked. Ataxia was more pronounced in the hind than the forelimbs, and was graded 1 and 2 out of 5 [29], respectively. Upper airway endoscopy including the guttural pouches revealed no abnormalities, based on the combination of clinical findings, intracranial disease was suspected.

A complete blood count and serum biochemistry profile showed a neutrophilia (8.13 × 109/L; reference interval [RI], 2.5–6), lymphopenia (1.19 × 109/L; RI 1.5–4), hypophosphataemia (0.44 mmol/L; RI 0.54–1.26), sideropenia (13.1 mmol/L; RI 15.2–40), and hyperglycaemia (7.03 mmol/L; RI 2.91–5.15). Lactate-dehydrogenase (LDH) (876 IU/L; RI 10–839) and sorbitol-dehydrogenase (SDH) (19 IU/L; RI 3–16) were also slightly increased. Adrenocorticotropic hormone (10.1 ng/L; RI < 30) and all other laboratory values (including serum amyloid A) were within normal limits.

Ultrasound-guided centesis of CSF between the first and the second cervical vertebra was performed as previously described [30]. CSF analysis revealed an increase in protein (0.82 g/L; RI 0.28–0.77) as the only abnormality.

## 3. Laboratory Work-Up for Infectious Diseases

To investigate several infectious agents potentially causing neurological disease in horses, blood and CSF samples were submitted for further analyses including real-time RT-PCR (qRT-PCR) for anaplasmosis (negative), serology for borreliosis (0.2 VE; RI < 8), qRT-PCR for borreliosis in the CSF (negative), serology for West Nile virus (WNV) (negative), and qRT-PCR (serum) for WNV (negative).

Serology for TBEV was performed by a private laboratory and revealed the following results: IgM 33.63 LE (RI < 25, negative; a modified version based of the human ELISA from Novatec was used, Novatec Immunodiagnostica GmbH, Dietzenbach, Germany) and IgG 381.70 U/mL (RI < 63, negative; Progen, Heidelberg, Germany). A qRT-PCR for TBEV in the CSF was negative. CSF IgM was 0.6 LE (no RI; to date not validated for CSF) and IgG 31 U/mL (RI < 20). Three different virus neutralization tests (VNTs) in CSF were performed to exclude cross-reacting antibodies with other relevant flaviviruses. The VNT was carried out under biosafety level 3 conditions using PK15-cells on 96-well plates. Test serum dilutions (20 μL starting serum material) were pre-incubated with 100 TCID50 of TBEV strain Neudoerfl (kindly provided by G. Dobler, Institute for Microbiology, Munich, Germany; GenBank accession no. U27495). All samples were run in duplicate and VNT titers were calculated 6 to 7 days after infection, depending on the cytopathic effects in the infected control wells. The neutralizing antibody titer, the neutralization dose 50% (ND50), of a serum was defined as the maximum dilution that inhibited cytopathic effects in 50% of the wells, and was calculated according to the Behrens–Kaerber method. ND50 values of above 10 were considered positive [28]. The VNT revealed the following results: TBEV ND50 1:60 (positive), West Nile virus (WNV) ND50 < 1:10 (negative), and Usutu-Virus (USUV) ND50 < 1:10 (negative). All results confirming an infection with TBEV are summarized in Table 1.

## 4. Treatment and Outcome

Treatment consisted of flunixine-meglumine (1.1 mg/kg IV BID for 3 days, then SID), prednisolone (2 mg/kg PO SID for 3 days, then 1 mg/kg PO SID), and vitamin E (5.5 mL PO SID). 

The horse remained hospitalized for six days and continued to show muscle fasciculations and periods of lethargy during which ataxia was still observed. The clinical signs gradually became less pronounced and less frequent and were absent by the end of the hospital stay on day 6. The horse was discharged with flunixine-meglumine (1.1 mg/kg PO SID), prednisolone (1 mg/kg PO SID), and vitamin E (5.5 mL PO SID) for another two weeks. 

Three weeks after discharge, the horse was presented for a re-examination. Vital parameters were within normal limits and the horse did not show any signs of lethargy according to the owner. The neurological examination was normal except for a slight persisting facial asymmetry with drooping of the nostril on the right side. 

Paired serum samples were collected 32 days after the onset of clinical signs, revealing the following results: TBEV IgM 2.00 LE (negative) and IgG > 500 U/mL (positive). The serum showed a neutralization titre of ND50 1:320 for TBEV-specific antibodies.

The owner reported that the gelding showed no signs of disease 12 months after discharge.

## 5. Discussion

Similar to the well-established situation in humans, recent serological (IgG in serum) and PCR-based surveys in horses [7] suggest that only few horses exposed to and infected with TBEV develop neurological signs. Nevertheless, increasing awareness and testing for WNV infection (and concomitant testing for TBEV) in Europe has also raised suspicion for TBEV-related neurological disease. However, to date, reports about ante-mortem diagnostic investigations of suspected cases are limited. Increasing awareness of the disease should encourage equine veterinarians to investigate suspected clinical cases according to the human diagnostic guidelines. Cases of TBE are hereby defined by the presence of clinical signs of meningitis, meningoencephalitis or meningoencephalomyelitis with CSF pleocytosis, the presence of specific TBEV serum IgM and IgG antibodies, CSF IgM antibodies, or TBEV IgG seroconversion [2]. This is the first report of a horse with suspected tick-bone encephalitis describing the laboratory work-up according to these guidelines. This case exhibited all of the above-described criteria, except for CSF pleocytosis.

Switzerland is considered free of WNV [31], whereas TBEV is endemic in large parts of Switzerland (www.bag.admin.ch; Accessed on: 1 July 2021). The area of origin of the horse (canton of Bern) described here is also known for the occurrence of TBE in humans. Clinical signs in horses after an infection with TBEV can range from subclinical or mild general illness [6,32] to severe neurological disease with fatal outcome [11,12,13]. However, most of the studies based their diagnosis on the measurement of serum antibodies [6,12,13]. The horse in this report showed clear signs of acute neurological disease and recovered with a slight asymmetry of the nostrils as the only remaining clinical signs after 4 weeks of anti-inflammatory and anti-oxidative treatment. Clinical signs described in another horse with suspected TBE with non-fatal outcome were non-specific such as impaired general health, reduced appetite, decreased body condition, nervousness, anxiety, rearing up, and unpredictability [6]. Diagnosis in this patient was based on a high TBEV-IgG serum titers (1:480 ND50), which decreased to 1:80 ND50 12 and 19 months later [6].

Laboratory findings in our patient were indicative of viral infection (neutrophilia and lymphopenia) and general inflammation (sideropenia). The other findings were non-specific and judged as not clinically relevant, although elevated liver enzymes have been described as a sequel of an infection with TBEV in humans [33,34,35]. In humans, the initial phase of TBE is reflected by leukopenia and/or thrombocytopenia, while the second (encephalic) phase is characterized by normal or mildly elevated leukocyte counts and normal platelet counts [36,37]. Similar findings have been described in dogs [38].

CSF analysis usually reveals pleocytosis [2], although there are reports of human patients with TBE without pleocytosis [39,40]. Accordingly, the absence of pleocytosis (as in our patient, which only showed elevated protein in CSF) does not rule-out TBE.

A TBEV qRT-PCR of the CSF was negative. Although direct identification of TBEV would be ideal for confirmation of the disease, serum and CSF qRT-PCR findings in affected humans are often negative. A possible explanation for negative qRT-PCR results in CSF is that the virus is not shed into the CSF, but spreads from cell to cell. This is supported by the fact that brain tissue is strongly positive in PCR [41]. Antibody tests are thus the recommended diagnostic choice to confirm an ante-mortem diagnosis [2]. A two-step method is recommended as described by Klaus et al., which includes a screening test with a modified ELISA and a subsequent confirmation of ELISA-positive cases by a serum neutralization test [14]. In our patient, IgM and IgG were positive in the first serum sample. CSF IgM and IgG levels were also assessed and the results are reported, although the method for IgM is not yet validated by the laboratory. Both CSF IgM and IgG were positive in this horse. In the second serum sample, IgM had returned to normal and IgG had increased, indicating recent acute infection. To rule out cross-reactivity with other flaviviruses, a serum neutralization test was performed confirming antibodies against TBEV and showing a five-fold increase (Table 1).

Other diagnostic modalities such as magnetic resonance imaging (MRI) have low sensitivity and specificity in the diagnosis of human TBE and are used to exclude other differential diagnoses [2]. An MRI under general anaesthesia was discussed in our patient, but this was declined by the owner.

Treatment is symptomatic in humans and animals as there is no antiviral treatment available for TBEV. The horse in this report responded well to a combination of anti-inflammatory drugs (flunixin-meguline, prednisolone) and anti-oxidative therapy (vitamin E), commonly used medications in equine patients experiencing viral neurological disease.

TBE prophylaxis is currently limited to reducing the contact to potentially infected ticks [10] in endemic areas and using repellents against tick infestation [14]. As Switzerland is considered an endemic area for TBEV infections (except for the two cantons Ticino and Geneva), those recommendations should be made for the whole country. There is no licensed vaccine available for use in horses to date. Although one report described the immunological response of a horse to human TBEV vaccine [14], further studies would be needed to evaluate safety and protection in horses [32]. Foals in endemic areas might be protected from TBE by passive transfer of immunity. One study found high TBEV antibody titers in 7-month-old foals in comparison with variable results in yearlings, suggestive of declining levels of antibodies following passive transfer [7].

## 6. Conclusions

In conclusion, this is the first report to describe ante-mortem diagnosis of TBE in a horse based on the diagnostic criteria recommended in human medicine. This diagnostic protocol may be implemented to investigate TBEV infection in horses living in endemic areas and showing compatible neurological signs. We recommend to implement this procedure in the diagnostic work-up of neurological cases with an uncertain etiology in combination with seasonal tick activity after ruling out a potential infection with WNV, even in countries that are yet considered free of WNV infections.

## Figures and Tables

**Table 1:** Serological and cerebrospinal fluid (CSF) analysis results of a horse with tick-borne encephalitis. The first sample was taken 1 day and the paired second sample 32 days after the onset of neurological signs.

Laboratory Findings	1st Sample	2nd Sample
**TBEV IgM ELISA (serum)**	33.63 LE	2.00 LE
**TBEV IgG ELISA (serum)**	381.70 U/mL	>500 U/mL
**TBEV VNT (serum)**	1 to 60	1 to 320
**TBEV PCR (CSF)**	negative	
**TBEV IgM ELISA (CSF)**	0.6 LE	
**TBEV IgG ELISA (CSF)**	31 U/mL	

## Data Availability

Not applicable.
